# Environmental filtering structures tree functional traits combination and lineages across space in tropical tree assemblages

**DOI:** 10.1038/s41598-017-00166-z

**Published:** 2017-03-09

**Authors:** Mengesha Asefa, Min Cao, Guocheng Zhang, Xiuqin Ci, Jie Li, Jie Yang

**Affiliations:** 1 Key Laboratory of Tropical Forest Ecology, Xishuangbanna Tropical Botanical Garden, Chinese Academy of Sciences, 666303 Yunnan, China; 2 University of Chinese Academy of Sciences, 100049 Beijing, China

## Abstract

Environmental filtering consistently shapes the functional and phylogenetic structure of species across space within diverse forests. However, poor descriptions of community functional and lineage distributions across space hamper the accurate understanding of coexistence mechanisms. We combined environmental variables and geographic space to explore how traits and lineages are filtered by environmental factors using extended RLQ and fourth-corner analyses across different spatial scales. The dispersion patterns of traits and lineages were also examined in a 20-ha tropical rainforest dynamics plot in southwest China. We found that environmental filtering was detected across all spatial scales except the largest scale (100 × 100 m). Generally, the associations between functional traits and environmental variables were more or less consistent across spatial scales. Species with high resource acquisition-related traits were associated with the resource-rich part of the plot across the different spatial scales, whereas resource-conserving functional traits were distributed in limited-resource environments. Furthermore, we found phylogenetic and functional clustering at all spatial scales. Similar functional strategies were also detected among distantly related species, suggesting that phylogenetic distance is not necessarily a proxy for functional distance. In summary, environmental filtering considerably structured the trait and lineage assemblages in this species-rich tropical rainforest.

## Introduction

The extraordinarily high plant diversity in the tropics has fascinated ecologists for decades. Various hypotheses have been proposed to explain the mechanisms that regulate the abundances and coexistence of different species^1, 2^. One of these is the habitat template hypothesis, which has gained considerable empirical support^3^. The habitat template hypothesis states that the habitat provides a template on which evolution shapes functional strategies and serves as an ecological filter to sort the natural histories of species at both spatial and temporal scales^4^. Different habitat templates have corresponding suites of biota with distinct ecological traits at the regional scale^5^. However, at the local scale, species in the community have evolved specific combinations of traits and life history characteristics that enable them to survive and depend on spatially specific environmental conditions^6^. Although studies examining the impact of habitat on species distributions date back many decades, the habitat template hypothesis has often been tested using traditional taxonomic-based approaches^7, 8^. These approaches may accurately describe spatial and temporal variation in species composition and structure under different environmental conditions. However, species names are relatively information-poor, and the species-centric approach therefore does not fully capture the mechanisms underlying species-environment relationships^9^. Hence, in order to understand how species in the community with certain traits and phylogenetic relationships persist under a defined set of environmental conditions, it is essential to explore beyond species per se.

Species distribution is constrained by abiotic and biotic filters through selection on their functional traits. Through functional traits, plants can shape, change, accommodate and follow the environment to fulfil their ecological requirements. McGill *et al.* ^10^ clearly illustrated the rebuilding of community ecology from functional traits to produce general principles. Trait-based ecology involves identifying the impacts of trait-mediated direct and indirect interactions among species in the community^11^. In this regard, the distribution of plant functional traits along environmental gradients has been investigated in different forest ecosystems^12–15^. As a consequence, each present-day species distribution is the result of ecologically relevant trait combinations that allow plants to persist in a defined set of environments^16^. Therefore, to compare plants’ functional strategies along different environmental gradients and to possibly infer assembly mechanisms, linking traits with filtering factors is very important.

The functional strategies of species can be quantified directly by the morphological traits of the species or indirectly using phylogenetic relatedness as a proxy for ecological similarity^17^. However, there is considerable debate about the relevance of phylogenetic information on trait-environment interactions. Species traits with no phylogenetic signal are filtered by the environment in a sub-tropical forest indicating evolutionary convergence (niche lability)^14^. On the other hand, evolutionary distance was indicated as a mediator for trait-environment relationships indicating phylogenetic niche conservatism^18^. Moreover, phylogenetic conservatism structured the assemblage of plant communities across environmental gradient^19^. To date, there have been no consistent reports on the importance of phylogeny in trait-environment relationships, and hence the dominant community assemblage mechanisms are still controversial. However, assessing the functional sorting of species by the environment without considering the importance of phylogenetic relationships for trait-environment association may not enable us to draw a comprehensive phylogenetically-based trait-environment conclusion on community assembly mechanisms. Therefore, it is essential to consider phylogenetic information on the trait-environment relationship to determine whether the functional strategies of species depend on evolutionary distance^18^.

Furthermore, the majority of studies have focused on the phylogenetic structure of communities to test the role of the environment in the distribution patterns of lineages^20^. Accordingly, phylogenetically clustered community structure has been taken as the most important evidence to verify the lineage-based effect of habitat filtering. However, very little research has quantitatively tested which lineages are filtered by the environmental factors in a specific geographical space^18^. This is more precisely to uncover how traits evolved among the lineages of the phylogeny. Therefore, phylogenetically-based community analysis in the absence of traits provides a partial view of community assembly mechanisms. This is because phylogenetically overdispersed communities can have either overdispersed or underdispersed traits that are conserved within lineages or converged across lineages respectively. Similar reasoning can be used for the phylogenetically clustered communities^21^, and hence the interpretation of community-level phylogenetic patterns depends on the distribution of ecologically relevant traits among the clades represented in the communities^22^. Therefore, it is imperative to integrate traits, phylogenies, and the environment across the geographical space to gain a complete understanding of the underlying community assembly mechanisms.

Phylogenetic community and trait structures have been found to be scale-dependent^23^. To the best of our knowledge, this is probably the first attempt to consider the trait-environment relationship within the geographical and phylogenetic contexts at different spatial scales. Parallel to this, the spatial scale could also affect trait and lineage dispersion patterns in plant communities. Previous studies in general have provided evidence that the roles of biotic and abiotic filters in structuring lineages and traits vary at different spatial scales, which can be verified by changing the spatial scale of the analysis^24, 25^. The co-existence of functionally similar or phylogenetically close species has been detected at a large spatial scale, while functionally and phylogenetically unrelated species co-occurrence has been observed at fine spatial scales^26, 27^. To this end, biotic interactions at a fine spatial scale and abiotic filtering at a large spatial scale were found to be respectively responsible for overdispersion and underdispersion of both phylogeny and traits^28^. Similarly, it has been indicated that due to the limiting similarity at fine spatial scale, co-existing species diverge both functionally and phylogenetically resulting in overdispersion. Scale-specific analysis could thus be effective for differentiating the competing mechanisms filtering trait and lineage dispersion across spatial scales^29^.

Here we analyse the relationships between traits and environmental gradients in phylogenetic and spatial contexts in a tropical forest dynamics plot in Xishuangbanna, China. We measured 11 plant functional traits and generated a molecular phylogeny for the 428 tree taxa in the plot. We used the ordination RLQ analysis^30^ and its extended version^18^ to integrate the analyses of traits, phylogeny, environment and space across four spatial scales (10 × 10 m, 20 × 20 m, 50 × 50 m, and 100 × 100 m). We assessed whether there is evidence of environmental filtering, and if present, we further identified the functional traits filtered by the environment at each spatial scale. We also identified and located the lineages affected by the filtering. Furthermore, we quantified the functional and phylogenetic dispersion of individual co-occurring trees along different environmental axes across the different spatial scales.

## Results

### Spatial autocorrelation

**Figure 1:**
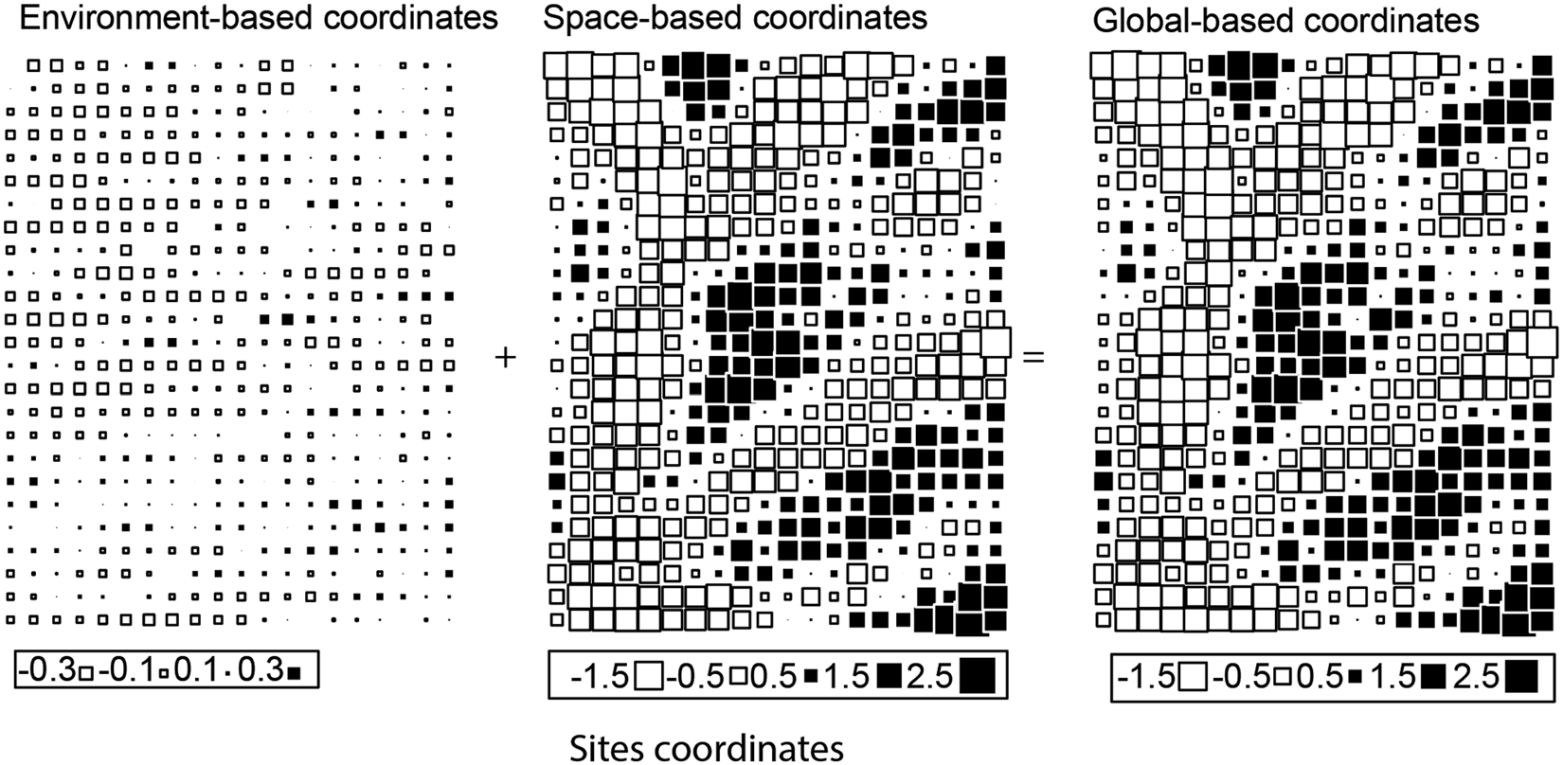
Results of the RLQ analysis visualized in geographic space at the 20 × 20 m spatial scale. The coordinates of sites are analysed on the first axis only. The global coordinates of the sites are defined as the sum of a combination of environmental variables and a combination of spatial variables. The size of the squares is proportional to the absolute values of the site coordinates; black and white squares indicate positive and negative coordinate respectively.

According to the Moran test, we found significant spatial autocorrelation in the environmental variables across the different spatial scales (Supplementary Table S1). The total nitrogen (TN), total phosphorus (TP), total potassium (TK), available nitrogen (AN), available phosphorus (AP), available potassium (AK), soil moisture, and elevation were significantly autocorrelated at all spatial scales. Carbon (C) and soil bulk density were significantly autocorrelated at small spatial scales (10 × 10 m and 20 × 20 m) but not at relatively large spatial scales (50 × 50 m and 100 × 100 m). In general, all environmental variables showed a clear environmental filtering gradient at all spatial scales (Fig. 1; Supplementary Figs S8 and S9).

### Correlation between traits and the environment

In general, we found more or less consistent significant associations between functional traits and environmental variables at all spatial scales except at 100 × 100 m (Supplementary Figs S2–S5). At the largest spatial scale (100 × 100 m), we did not detect significant correlation between functional traits and environmental variables, and we thus excluded this spatial scale from further analysis (Supplementary Fig. S5). The first axis of the RLQ applied to both space and the environment, and both traits and phylogeny, explained 34.1%, 41.21%, and 43.56%, of the total variation across the 10 × 10 m, 20 × 20 m, and 50 × 50 m spatial scales, respectively.

**Figure 2:**
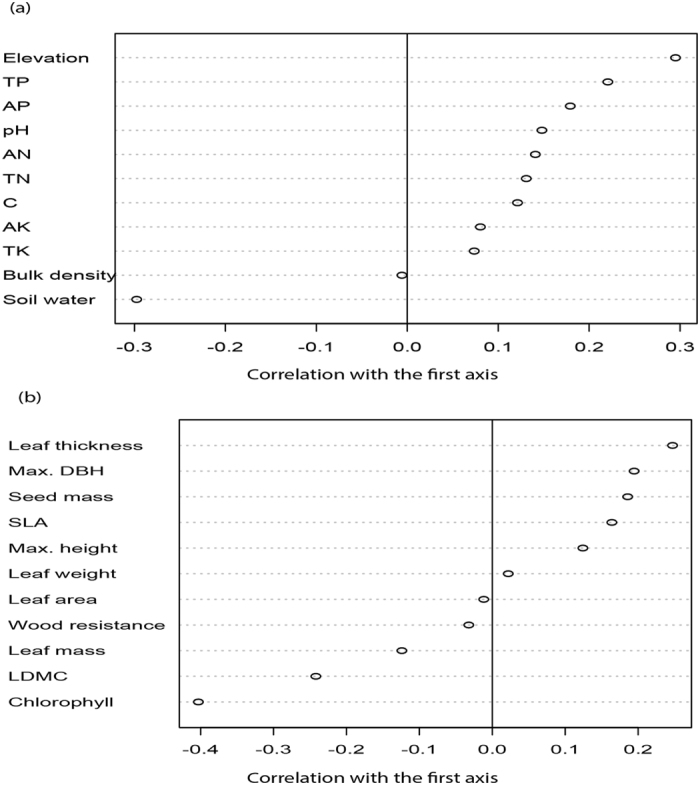
Effects of environmental variables and functional traits on the first axis of the RLQ analysis at the 20 × 20 m spatial scale. (**a**) Pearson correlation between environmental variables and the coordinates of sites on the first axis. (**b**) Pearson correlation between traits and the coordinates of species on the first axis. TN, total nitrogen; TP, total phosphorus; TK, total potassium; AN, available nitrogen; AP, available phosphorus; AK, available potassium; C, carbon; Max. DBH, maximum diameter at breast height; SLA, specific leaf area; LDMC, leaf dry matter content.

At the 10 × 10 m spatial scale, species with high leaf thickness, seed mass, maximum height, and leaf weight were positively correlated with high soil nutrient contents in the plot, whereas, species with high leaf dry matter content (LDMC), high wood resistance, high chlorophyll content, and high leaf area were correlated with the negative side of the RLQ axis which represented areas with limited soil nutrients but high soil water content (Supplementary Fig. S6). At the 20 × 20 m and 50 × 50 m spatial scales, the nutrient-rich part of the plot is occupied by species with high specific leaf area, maximum height, maximum diameter at breast height, leaf thickness, seed mass, leaf weight, and leaf area, while, species with high leaf dry matter content (LDMC), high wood resistance, high chlorophyll content, and high leaf mass were associated with the nutrient-limited part of the plot (Fig. 2; Supplementary Fig. S7).

### Relationships among traits, phylogeny, environment, and space

The global fourth-corner analysis showed that the association of the environmental and spatial variables with both the biological traits and phylogenetic variables were significant at all scales. The global fourth-corner test on both space and the environment, and on both traits and phylogeny, was significant with the observed value far from the theoretical values (P < 0.05). The associations of the environment with traits, the environment with phylogeny, space with traits, and space with phylogeny were all significant (Table 1).

**Table 1:** Summary of the fourth-corner tests for the association between environmental (E), spatial (S), trait (T) and phylogenetic (P) matrices at different spatial scales.

Spatial scales (m^2^)	Pair of matrices	Obs	SES	P
10 × 10	R and Q	0.013	3.42	0.042
	E and T	0.003	4.13	0.036
	E and P	0.005	2.52	0.034
	S and T	0.152	3.42	0.001
	S and P	0.08	2.31	0.001
20 × 20	R and Q	0.026	3.03	0.023
	E and T	0.021	5.52	0.033
	E and P	0.036	4.32	0.013
	S and T	0.122	2.41	0.015
	S and P	0.162	3.35	0.003
50 × 50	R and Q	0.034	2.02	0.030
	E and T	0.031	2.11	0.021
	E and P	0.121	5.23	0.023
	S and T	0.101	4.33	0.028
	S and P	0.103	3.54	0.011

Obs, observed value; SES, standardized effect size.

### Lineage distribution

**Figure 3:**
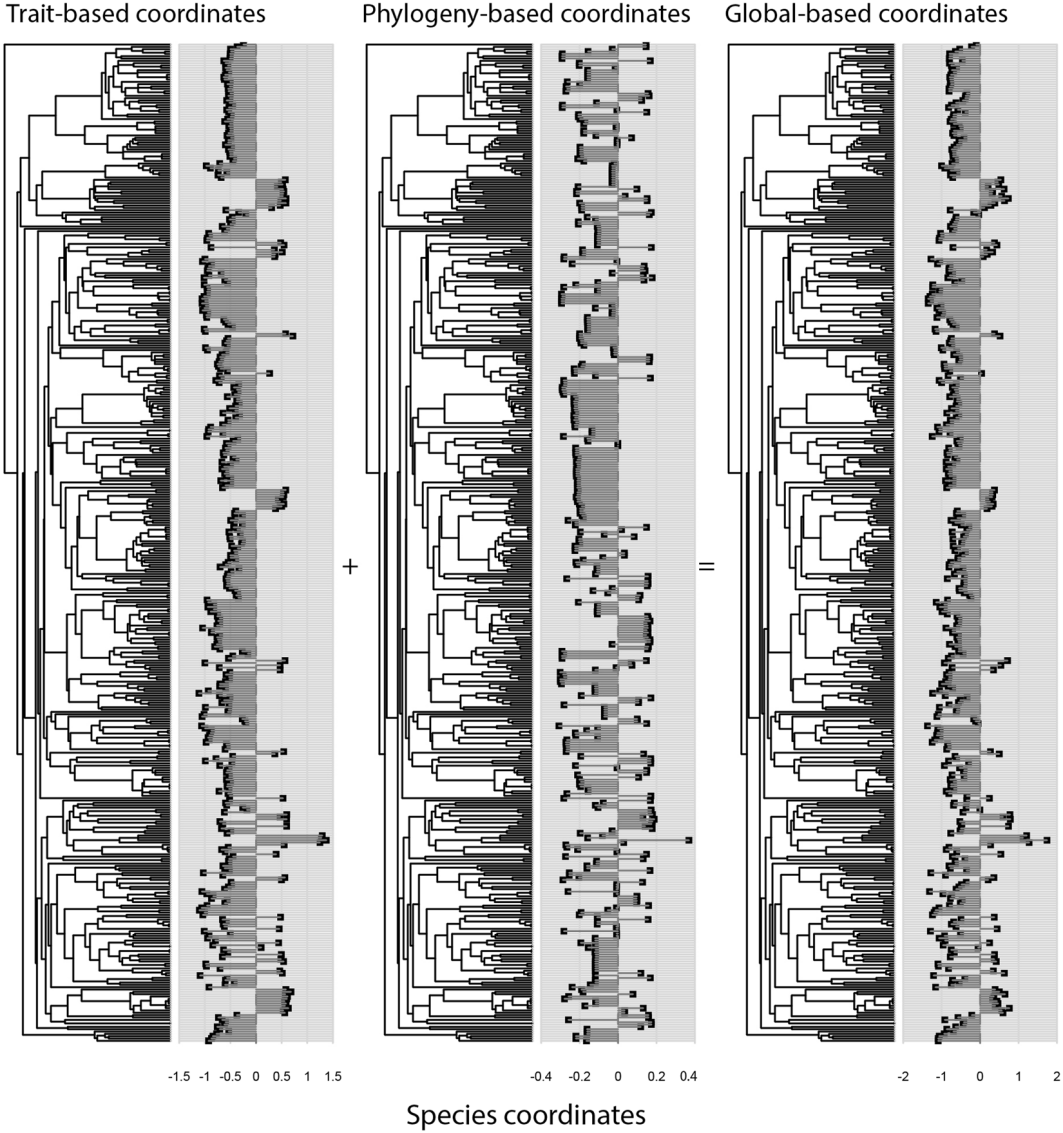
Results of the RLQ analysis visualized on the phylogeny at 20 × 20 m. The coordinates of species are analysed on the first axis only. The global coordinates of the species are defined as the sum of a combination of trait variables and a combination of phylogenetic variables.

The distribution of lineages varied with environmental variables across the different spatial scales. The areas with high soil nutrient contents were particularly dominated by the Euphorbiaceae (such as *Epiprinus siletianus*) and Annonaceae (such as *Polyalthia simiarum*) families at small spatial scales (10 × 10 m and 20 × 20 m) (Fig. 3; Supplementary Fig. S10). There were also species in the Moraceae and some species in the Rubiaceae and Icacinaceae families associated with high soil nutrient contents at the 50 × 50 m scale (Supplementary Fig. S11). There were also species associated with the relatively infertile part of the plot, particularly *Pittosporopsis kerrii* (Icacinaceae) at the 20 × 20 m scale (Fig. 3). Additionally, at the larger scale, species both in the Lauraceae and Fagaceae families were also found in regions with relatively limited soil nutrients (Supplementary Fig. S11).

### Phylogenetic and trait dispersion pattern

Non-random functional and phylogenetic dispersion patterns were found for the respective positive and negative sides of the RLQ axes across the four studied spatial scales (Student’s t-test, *P* < 0.05). We detected both functional and phylogenetic clustering for each spatial scale and environmental axis, with the strength of clustering lower in phylogenetic dispersion than in functional dispersion (Table 2).

**Table 2:** Phylogenetic and functional dispersion patterns at three spatial scales.

Spatial scales (m^2^)	Functional dispersion (S.E.S. PW)	Phylogenetic dispersion (NRI)
**Positive axis**		
10 × 10	0.012	0.023
20 × 20	0.103	0.987
50 × 50	2.087	2.221
**Negative axis**		
10 × 10	3.211	2.142
20 × 20	3.098	2.004
50 × 50	1.245	1.358

## Discussion

We used functional traits, phylogenetic, environmental, and spatial data to explore phylogenetically-based trait distribution patterns across an environmental gradient at different spatial scales. The extended version of the RLQ approach was applied to quantify the effect of an environmental gradient on trait and lineage distributions across multiple spatial scales in a tropical forest. Our research demonstrated that environmental filtering considerably structured the functional strategies of plants in a species-rich tropical rainforest.

### Spatial autocorrelation in the environmental factors

The spatial autocorrelation result showed that the distributions of a majority of the environmental variables were significantly affected by geographical space at all spatial scales (Fig. 1; Supplementary Figs S8 and S9). A clear environmental gradient was observed in the plot at all spatial scales, which act as an environmental filter. As a result, significant associations of most of the functional traits with the environmental gradient were detected across all spatial scales except at 100 × 100 m (Supplementary Figs S2, S3, S4, and S5). The absence of a significant correlation between traits and the environment at the 100 × 100 m scale could be attributed to the heterogeneity of habitats. A wide range of environmental conditions exist as the spatial scale increases, and as a consequence, there might not be a significant specific linkage between functional traits and the environment, perhaps suggesting a high niche-breadth of species.

Additionally, our global fourth-corner analysis also showed that the associations between the environment and phylogeny, the environment and traits, space and traits, and space and phylogeny were significant at all scales (Table 1). This suggests that the observed distributions of traits and lineages are not the result of random processes in tropical tree assemblages, and hence the RLQ axes represent a relevant dimension of differentiation.

### Relationships among traits, phylogeny, environment, and space

At all spatial scales (10 × 10 m, 20 × 20 m, and 50 × 50 m), in general, the variation in soil nutrient availability in space determined the lineage distribution and range of the functional strategies of species. Species with resource acquisition-related traits were associated with the nutrient-rich part of the plot, whereas, the nutrient-limited part of the plot was occupied by the species with resource conservation-related traits. Specifically, species with high specific leaf area, leaf thickness, leaf weight, maximum height, and maximum diameter at breast height (max. DBH) were distributed in the resource-rich part of the plot (Fig. 2). This result is possibly consistent with the idea that plant species in a resource-rich environment are characterized by both fast growth and a high mortality rate^31^. Our finding is in agreement with the trait-soil relationship reported in a tropical rainforest in the Lambir forest dynamics plot^12^ and in a sub-tropical evergreen broad-leaved forest^31^. Plant species in such environments are expected to produce structurally cheap leaves with high specific leaf area that can photosynthesize at a faster rate, and structurally cheap wood permitting fast volumetric growth into the canopy with a maximum height to acquire sufficient solar radiation. Our results clearly showed that the filtering of species by soil nutrient availability was related to their phylogenetic position. Regions with high soil nutrient content are particularly dominated by the Euphorbiaceae (such as *Epiprinus siletianus*) and Annonaceae families (such as *Polyalthia simiarum*). There are also species distributed in the nutrient-limited part of the plot, such as *Pittosporopsis kerrii* (Icacinaceae) (Fig. 3).

Species that prefer limited resource environments are ecologically strategic, promoting structural investment at the cost of reduced resource acquisition and demographic rates^31^. In this regard, at all spatial scales (10 × 10 m, 20 × 20 m, and 50 × 50 m), we found species with high wood resistance, LDMC, chlorophyll content, leaf area, and leaf mass associated with the resource-limited part of the plot. The low availability of nutrients in this environment could be linked to the high acidity level of the soil, which has also been reported by John *et al.* ^32^. The presence of species with high wood resistance in this part of the plot might be related to their low growth rate, and estimated to have smaller vessels and thicker fibre walls^33^. Similarly, species with high wood resistance were found to be negatively correlated with the nitrogen and phosphorus content^34^ as well as soil fertility in the BCI plot^31^. Species with low wood resistance have also been distributed in resource-rich environments in a sub-tropical forest^31^. High LDMC species were also found in the resource-limited environment in our plot, which may provide further resistance to physical hazards such as herbivory, wind, and hail to maximize their life span^35^.

However, plants in the nutrient-limited region contained high leaf chlorophyll content, which may be a physiological response to low light environments given they are short and restricted to low elevations (at 10 × 10 m and 20 × 20 m). In this part of the plot, the elevation is low, and light could be therefore a limited resource for plant growth and survival^36^. In this nutrient-limited part of the plot, plants are generally characterized by a large leaf area, which is consistent with the findings of Yan *et al.* ^37^. However, this pattern contradicts the concept that plants with conservative leaf strategies are found to be mostly distributed in resource-limited environments and characterized by a small leaf size^38^, as a large leaf size is usually associated with high resource acquisition and growth rates^31^. Furthermore, it has also been documented that dry conditions might select small-leaved plants^39^. Accordingly, our results indicate that large-leaved species are restricted to soil high in water content, whereas small-leaved plants were mainly found in parts of the plot with low soil water content.

Seed mass is one of the fundamental seed traits linked with species regeneration capacity. In environments with high nutrient supplies, it is expected that a large number of smaller seeds can be produced rapidly, and hence species with a low seed mass might have the advantage of high dispersal ability, germination, and rapid growth^40^. However, at all spatial scales (10 × 10 m, 20 × 20 m, and 50 × 50 m), perhaps unexpectedly, we found that species with a large seed mass preferred the resource-rich part of the plot, while small-seeded species were distributed in the nutrient-limited part of the plot. A similar report also showed that large seed mass associated with nutrient-rich environments^41^. In summary, variation in the soil resource availability at these scales shapes the distribution of functional traits in a tropical forest.

### Phylogenetic and trait dispersion pattern

Apart from the effect of environmental filtering through the link between the environment and traits, we also examined phylogenetic and trait dispersion patterns. Our results indicate that species could also be structured based on phylogenetic and functional similarity at all spatial scales (Table 2). The detection of phylogenetic clustering in our plot is consistent with the hypothesis of phylogenetic niche conservatism^42^. Accordingly, our work revealed that phylogenetically clustered species appear to share similar ecological requirements (Fig. 3). Tanentzap and Lee^19^ also indicated that species are distributed based on their evolutionary relationship along the flooding gradient. Yang *et al.* ^28^ tested the phylogenetic signal in functional traits in our plot that has been used to explain the importance of phylogenetic distance among species as predictors of functional distance^20^. Consequently, functional traits were indicated to have a weaker phylogenetic signal than expected by chance. Even if the degree of phylogenetic conservatism was not as strong as expected in the results of Yang *et al.* ^28^, our results indicate that the phylogenetic signal might also roughly shape the structure of traits in space since closely related species were found to share similar functional strategies (Fig. 3). Our results are consistent with the findings of Pavoine *et al.* ^18^ showing the clustering of lineages and traits. However, in contrast to our results, phylogenetic overdispersion has also been documented^25^, which supports the phylogenetic limiting similarity hypothesis. Similarly, significant phylogenetic overdispersion at small spatial scales was also reported by Swenson *et al.* ^43^, supporting competitive exclusion.

Despite the fact that we did find lineage clustering, we should not ignore the fact that our results also indicate that distantly related species were also distributed in the same environment, showing that they have similar ecological requirements (such as Euphorbiaceae and Annonaceae preferring nutrient-rich environments). This may be linked with the weak phylogenetic signal of traits^28^. Our finding is similar to the reports of Pavoine *et al.* ^18^ showing that distantly related species share similar functional strategies. Because of the evidence for trait convergence, the evolutionary distance among species might not be sufficient to explain species distributions across nutrient gradients, and as a result, phylogenetic distance essentially cannot always be used as a proxy for functional distance.

## Conclusions

Environmental filtering was detected at all spatial scales, except 100 × 100 m, and this filtering considerably structured the functional strategies of lineages in a species-rich tropical rainforest. We also found that in general, spatial variation in soil nutrient availability determined the range of the ecological strategies of species. Species with high resource acquisition-related traits were primarily associated with the resource-rich part of the plot, whereas resource conservation functional traits that enable species to follow a resource conservation strategy were distributed in limited-resource environments. Furthermore, both phylogenetic and functional clustering were detected at all spatial scales, implying that the observed distributions of traits and lineages in space were not the result of random assembly processes. We also found that both trait conservatism and trait convergence shape the functional strategies of species, perhaps suggesting that the distribution of functional traits in space cannot necessarily be linked with the evolutionary distances of species.

## Methods

### Study area

This study was conducted in the 20-ha Xishuangbanna Forest Dynamics Plot (FDP) (21°36′N, 101°34′E) in Yunnan province, southwest China (Supplementary Fig. S1). The plot is located in Xishuangbanna National Nature Reserve and is characterized by rugged topography with mountain ridges running from north to south showing a marked altitudinal gradient. The topography of the plot is complex with an elevational range from 709 m to 869 m. The highest elevation is located in the northwest part of the plot. The plot is defined by three perennial streams that join together at the southwest part of the plot^44^. The soil of Xishuangbanna is characterized by laterite, lateritic red soil, and limestone-derived soil. These soil types are delimited by elevation. The soil of the Xishuangbanna tropical seasonal rainforest, in which the plot is located, is characterized by laterite soil with a pH range of 4.5–5.5 ^45^. The Xishuangbanna FDP occurs in a seasonal tropical rainforest and is dominated by large individuals of *Parashorea chinensis* (Dipterocarpaceae). Our first census was conducted in 2007, and all freestanding woody stems ≥1 cm in diameter at 130 cm from the ground (diameter at breast height, DBH) were measured, mapped, tagged, and identified to the species level^44^. We used these data for our analyses.

### Environmental data

We used soil nutrients as environmental factors in our analysis. Soil samples were collected and analysed for the Xishuangbanna FDP following the standardized protocols described in John *et al.* ^32^. Specifically, soil was sampled using a regular grid of 30 × 30 m in the 20-ha plot. The 252 grid intersections were basal collection points:-, together with each base point, three additional sampling points were located at random combinations of 2 and 5 m, 2 and 15 m or 5 and 15 m along a random compass bearing away from the associated base point. At each sampling point, we removed the litter and humus layer, and then collected 500 g of topsoil from a depth of 0–10 cm. A total of 756 soil samples were taken. The fresh soil samples were placed into pre-labelled plastic bags and shipped to the Biogeochemistry Laboratory at the Xishuangbanna Tropical Botanical Garden. In the laboratory, one sub-sample was used to measure pH values as immediately as possible using a potentiometer in fresh soil after water extraction (soil: water was 1: 2.5). The other sub-samples were air-dried, smashed, sieved using 1 mm and 0.15 mm mesh and stored in plastic bags for later additional analyses^46^. The volumetric soil water content (%) was measured in the late dry season and late rainy season from 2011 to 2015, using the mean values of three replicates taken randomly from around the centre of each seedling quadrat using a TDR probe (MPM-160B) at a depth of 5 cm.

The soil bulk density was measured using the corer method, and the soil organic matter was measured in soil oxidized with H_2_SO_4_-K_2_Cr_2_O_7_. We used the Walkley-Black method to estimate the carbon content (C). The micro-Kjeldahl method was used to evaluate the total nitrogen (TN), and micro-diffusion was used to determine the available nitrogen (AN) in the soil. The soil was digested in an HNO_3_-HClO_4_ solution, and the total phosphorus (TP) and potassium (TK) were determined using an inductively coupled plasma atomic emission spectrometer. The extractable phosphorus (AP) was released from the soil in a solution containing 0.03 mol L^−1^ NH_4_F and 0.025 mol L^−1^ HCl and estimated colorimetrically. Exchangeable potassium (AK) was extracted in a neutral 1-mol L^−1^ CH_3_COONH_4_ solution, and the TK in the extract was determined using an inductively coupled plasma atomic emission spectrometer. Finally, we obtained spatial maps of the elevation, volumetric soil water content and 9 soil nutrients (pH, TN, TP, TK, AN, AP, AK, C, and bulk density) in each plot. Additional detailed information regarding the soil data collection can be found in Hu *et al.* ^46^.

It has been demonstrated that the role of habitat filtering is dependent on the spatial scale and is more prevalent at the mesoscale level^28^. Therefore, to test the relative effect of habitat filtering on functional traits and lineages at different spatial scales, we divided the plot into square quadrats with different areas: 10 × 10 m (n = 2000), 20 × 20 m (n = 500), 50 × 50 m (n = 80), and 100 × 100 m (n = 20).

### Trait selection and measurement

Trait data for the tree species in the plots were obtained from vegetation samples collected using standardized protocols^39^. In total, 11 functional traits were selected based on the expectation that these traits represent the basic functional trade-offs in leaves, wood, and seeds among tree species^38^. We measured leaf area (cm^2^), specific leaf area (SLA, cm^2^.g^−1^), leaf chlorophyll content (g.cm^−1^), leaf thickness (mm), leaf mass, leaf weight, leaf dry matter content (LDMC), seed dry mass (g), wood specific resistance (N), the maximum tree height (m), and the maximum tree diameter at breast height (cm).

We quantified leaf traits from randomly collected mature outer canopy leaf samples from adult trees. Usually, outer the canopy leaves were sun leaves collected from individuals that were ten metres tall. However, in the case of small, shade-tolerant species, the outer canopy leaves were necessarily shade leaves collected from individuals less than ten metres tall, given that these species do not occupy gaps or reach the forest canopy. We collected more than five leaves from each of five individuals of each species belonging to 428 taxa by making sure that the collected leaves did not have any obvious symptoms of pathogen or herbivore attack or substantial cover of epiphylls^39^. For rare taxa, the data on functional traits were collected outside of the plots. We measured wood-specific resistance using a Resistograph on the five largest individuals of each of 420 taxa. These traits possibly vary across size-classes within species, potentially limiting our ability to learn about community assembly via adult traits. Nevertheless, interspecific variation in traits may be largely consistent across ontogeny, and traits measured on adults often correspond to major axes of niche, demographic, and life history variation among species. Here, we focus on adult traits as predictors of demography across different life stages. The mean trait value for each species was used in all analyses.

### Phylogenetic tree reconstruction

We reconstructed a phylogenetic tree for the Xishuangbanna FDP representing 428 taxa. Using three chloroplast sequence regions – rbcL, matK, trnH-psbA and the nuclear ribosomal internal transcribed spacer (ITS), a DNA supermatrix was generated^47^. Based on the method described in Kress *et al.* ^48^, the rbcL and matK regions were globally aligned. The trnH-psbA and ITS regions were aligned within families using the software package SATé^49^ and then concatenated to the rbcL and matK alignments. The DNA supermatrix was then analysed using RA × ML^50^ via the CIPRES supercomputer cluster^51^ to infer a maximum likelihood (ML) phylogeny using the APG III phylogenetic tree as a constraint or guide tree^48^. A constraint tree approach helps to ensure that the basal topology of a molecular community phylogeny is consistent with the global working hypothesis for the angiosperm basal topology. Node support was estimated using bootstrap values with nodes with less than 50% support being collapsed into soft polytomies. Finally, an ultrametric tree was obtained using the non-parametric rate smoothing approach in the r8s software package^52^.

#### Data analysis

To obtain normally distributed data, we transformed both the environmental and trait data using a natural log transformation, and checked these data for correlations. To further illustrate which lineages and combinations of functional trait states are filtered by the environment in an explicitly spatial context, we used the extended RLQ approach proposed by Pavoine *et al.* ^18^. The spatial variables in our analyses are based on the 20 × 20 m quadrat, and the environmental variables contained 10 soil parameters (pH, TN, TP, TK, AN, AP, AK, C, bulk density, soil water). The RLQ approach was originally developed by Dolédec *et al.* ^30^. The extended RLQ approach proposed by Pavoine *et al.* ^18^ contains five matrices: an environmental matrix (E), a spatial matrix (S), a phylogenetic distance matrix (P), a functional trait distance matrix (T), and a species-by-sites matrix (L). The four matrices (E, S, P, and T) are linked by the matrix L, whose rows represent sites, and columns contain species abundances. Before the extended RLQ analysis, we tested the correlations between functional traits and environmental variables using the extended version of the fourth-corner approach and applied the combination of null model 2 and model 4^53^. Accordingly, we included traits significantly associated with the environment in the extended RLQ analysis. Similarly, the analysis of the significance of the connections between matrices E and P, S and T, and S and P was carried out using this approach. To prepare the matrices for the extended RLQ analysis, all matrices were analysed separately with different ordinations. First, the species-by-site matrix (L) was analysed using a canonical analysis. The environmental matrix E was analysed by principal component analysis (PCA). The spatial matrix S was defined as the eigenvectors of a neighbour matrix and was analysed using the PCA approach. The trait distance matrix T and phylogenetic distance matrix P were analysed by principal coordinate analysis (PCoA)^18, 30^.

The extended RLQ analysis combines the five separate ordination analyses to maximize the co-variation between the environmental variables and traits or phylogeny, spatial variables and traits or phylogeny with the use of co-inertia analysis. Within the given constraints, it selects the axis that maximizes the covariance in the L matrix, resulting in a compromise between the best joint combination of site scores by their environmental and spatial characteristics, the best combination of species scores by their trait and phylogeny attributes and the simultaneous ordination of sites and scores^30, 54^. The extended RLQ analysis was implemented using the R codes provided by Pavoine *et al.* ^18^ based on the packages “spdep”^55^ and “ade4”^56^.

To test the influence of environmental factors at different spatial scales, we applied the null model to determine whether the observed phylogenetic and trait dispersion patterns are different from those expected in the environment based on random chance. Accordingly, given a tree topology and branch lengths, we randomly shuffled the names of taxa across the tips of the phylogeny and trait dendrograms 999 times. This null model randomizes the relatedness of species with one another while maintaining species abundance, frequency, and occurrence. A null phylogenetic or traits diversity value was produced from each iteration. The 999 null values constituted a null distribution to which the observed values were compared. A net relatedness index (NRI) was used to quantify the phylogenetic dispersion of co-existing species. This is the standardized effect size (S.E.S.) for the mean pairwise phylogenetic distance (MPD) for all individuals in each quadrat, which is the abundance-weighted calculation relative to the previous calculation^20, 43^. The functional dispersion of the species was determined using a standardized effect size (S.E.S.) PW for each spatial scale. The NRI and S.E.S. PW were calculated using the following equations:

1 $$NRI=-1\times \frac{(MP{D}_{obs}-mean(MP{D}_{null}))}{sd(MP{D}_{null})}$$

2 $${S}{.E}{.S}{.}\,{PW}=-1\times \frac{(P{W}_{obs}-mean(P{W}_{null}))}{sd(P{W}_{null})}$$

where, *MPD* is the mean pairwise phylogenetic distance between all individuals within a local sample. *PW* is the mean pairwise trait distance from the functional trait dendrogram for all individuals within a local sample. *MPD*_*obs*_ and *PW*_*obs*_ represent the observed value of the mean pairwise phylogenetic/traits distances. The *mean*(*MPD*_*null*_) and *mean*(*PW*_*null*_) represent the mean values from a null distribution in which species names were randomly shuffled 999 times on the tips of the community phylogeny or trait dendrogram, and the *MPD* and *PW* values were calculated each time for each quadrat. The *sd*(*MPD*_*null*_) and *sd*(*PW*_*null*_) represent the standard deviations of the null distribution. A positive *NRI* or *S*.*E*.*S*. *PW* indicates that species in the environment are phylogenetically or functionally clustered, whereas a negative *NRI* or *S*.*E*.*S*. *PW* indicates that an assembly is phylogenetically or functionally overdispersed respectively. The tip shuffling null model was used as it only randomizes relatedness and does not randomize any spatial or size data. Therefore, it enables us to ask and address specific hypotheses regarding whether relatedness alone is non-random while fixing the observed spatial patterns in the assemblages.

The phylogenetic and functional traits dispersion values were calculated by repeating the analysis for each spatial scale: 10 × 10 m, 20 × 20 m, 50 × 50 m, and 100 × 100 m. We used simultaneous spatial autoregression (SAR) because the *NRI* and *S*.*E*.*S*. *PW* values in the quadrats were spatially autocorrelated, and this method uses neighbour matrices to estimate spatially independent data points that can be utilized for analysis. Furthermore, we used a generalized least-squares model with a first-order spatial neighbour SAR using “spdep” package^57^ in R. To test for significant deviations of *NRI* or *S*.*E*.*S*. *PW* from the expectation of zero, we applied Student’s t-test. The analyses were implemented in R^58^ using the packages “picante”^59^, “gstat”^60^, and “ade4”^56^.

## Electronic supplementary material

Supplementary information
