# Supplementary material for: Environmental filtering structures tree functional traits combination and lineages across space in tropical tree assemblages
Source: Sci Rep. 2017 Mar 9;7:132. doi: 10.1038/s41598-017-00166-z (PMC5427853; doi:10.1038/s41598-017-00166-z)
Supplement: Supplementary file 1 — Supplementary information [file 41598_2017_166_MOESM1_ESM.pdf]

Environmental filtering structures tree functional traits combination and lineages across space in tropical tree assemblages

Mengesha Asefa[1,2], Min Cao[1], Guocheng Zhang[1], Xiuqin Ci[1], Jie Li[1], Jie Yang[1#]

[1]Key Laboratory of Tropical Forest Ecology, Xishuangbanna Tropical Botanical Garden, Chinese Academy of Sciences, 666303, Yunnan, China

[2]University of Chinese Academy of Sciences, 100049, Beijing, China

[#]corresponding author: Jie Yang, yangjie@xtbg.org.cn

Figure legends

Figure S1 Geographical location map of Xishuangbanna forest dynamics plot (The black circle dot). The map was generated using ArcGIS 10.1 (www.esri.com).

Figure S2 Correlation of functional traits with the first two axes of environmental gradients using combination of fourth corner and RLQ results at the 10 × 10 m spatial scale. Significant (P, 0.05). Red cells indicate positive significant associations, blue cells indicate significant negative associations. Grey cells indicate non-significant associations.

Figure S3 Correlation of functional traits with the first two axes of environmental gradients using combination of fourth corner and RLQ results at the 20 × 20 m spatial scale. Significant (P, 0.05). Red cells indicate positive significant associations, blue cells indicate significant negative associations. Grey cells indicate non-significant associations.

Figure S4 Correlation of functional traits with the first two axes of environmental gradients using combination of fourth corner and RLQ results at the 50 × 50 m spatial scale. Significant (P, 0.05). Red cells indicate positive significant associations, blue cells indicate significant negative associations. Grey cells indicate non-significant associations.

Figure S5 Correlation of functional traits with the first two axes of environmental gradients using combination of fourth corner and RLQ results at the 100 × 100 m spatial scale. Significant (P, 0.05). Red cells indicate positive significant associations, blue cells indicate significant negative associations. Grey cells indicate non-significant associations.

Figure S6 Effects of environmental variables and functional traits on the first axis of the RLQ analysis at the 10 × 10 m spatial scale. (a) Pearson correlation between environmental variables

and the coordinates of sites on the first axis. b) Pearson correlation between traits and the coordinates of species on the first axis.

Figure S7 Effects of environmental variables and functional traits on the first axis of the RLQ analysis at the 50 × 50 m spatial scale. (a) Pearson correlation between environmental variables and the coordinates of sites on the first axis. b) Pearson correlation between traits and the coordinates of species on the first axis.

Figure S8 Result of the RLQ analysis visualized on the geographic area at 10 × 10 m. The coordinates of sites analysed on the first axis only. The global coordinates of the sites are defined as the sum of a combination of environmental variables and a combination of spatial variables. The size of the squares is proportional to the absolute values of the site coordinates; black squares indicate a positive coordinate, and white indicate a negative coordinate.

Figure S9 Result of the RLQ analysis visualized on the geographic area at 50 × 50 m. The coordinates of sites analysed on the first axis only. The global coordinates of the sites are defined as the sum of a combination of environmental variables and a combination of spatial variables. The size of the squares is proportional to the absolute values of the site coordinates; black squares indicate a positive coordinate, and white indicate a negative coordinate.

Figure S10 Result of the RLQ analysis visualized on the phylogeny at 10 × 10 m. The coordinates of species are analysed on the first axis only. The global coordinates of the species are defined as the sum of a combination of trait variables and a combination of phylogenetic variables.

Figure S11 Result of the RLQ analysis visualized on the phylogeny at 50 × 50 m spatial scale. The coordinates of species are analysed on the first axis only. The global coordinates of the species are defined as the sum of a combination of trait variables and a combination of phylogenetic variables.

Table S1 Spatial autocorrelation of environmental variables across different spatial scales.

Spatial scales (m^2)	Test	Obs	Std. obs	Alter	P-value
10 × 10	Elevation	0.9886426	86.40427	Greater	0.001
	TN	0.2688364	23.97544	Greater	0.001
	TP	0.2462586	21.33255	Greater	0.001
	TK	0.2219802	19.52048	Greater	0.001
	AN	0.2647110	23.92373	Greater	0.001
	AP	0.2683324	22.26044	Greater	0.001
	AK	0.2757978	22.84013	Greater	0.001
	C	0.2695628	23.90139	Greater	0.001
	pH	0.1380615	12.38516	Greater	0.001
	Bulk density	0.2910171	24.77080	Greater	0.001
	Soil water	0.7817030	66.02583	Greater	0.001
20 × 20	Elevation	0.96449899	42.761044	Greater	0.001
	TN	0.34677233	14.823621	Greater	0.001
	TP	0.45021279	19.925771	Greater	0.001
	TK	0.36619529	16.292482	Greater	0.001
	AN	0.22895160	10.135292	Greater	0.001
	AP	0.27738989	12.352112	Greater	0.001
	AK	0.25778863	11.417491	Greater	0.001
	C	0.16497258	7.087407	Greater	0.001
	pH	0.32600659	14.121632	Greater	0.017

	Bulk density	0.05152216	2.346026	Greater	0.001
	Soil water	0.73372623	32.724127	Greater	0.001
50 × 50	Elevation	0.86908983	15.5250258	Greater	0.001
	TN	0.31246803	5.4700108	Greater	0.001
	TP	0.36893419	6.3241412	Greater	0.001
	TK	0.32588612	5.4169539	Greater	0.001
	AN	0.16500617	2.9328830	Greater	0.005
	AP	0.16342192	2.9555149	Greater	0.005
	AK	0.25639173	4.5265385	Greater	0.001
	C	0.03840909	0.8459071	Greater	0.201
	pH	0.25476748	4.6094408	Greater	0.001
	Bulk density	-0.10766035	1.7187902	Greater	0.967
	Soil water	0.84572684	14.2351490	Greater	0.001
100 × 100	Elevation	0.6136811	6.341305	Greater	0.001
	TN	0.5388617	5.148104	Greater	0.001
	TP	0.3974735	3.869749	Greater	0.002
	TK	0.3214555	3.584413	Greater	0.004
	AN	0.4530939	4.353877	Greater	0.001
	AP	0.2643541	2.797702	Greater	0.010
	AK	0.4505024	4.403740	Greater	0.001
	C	0.4694378	4.625534	Greater	0.201
	pH	0.1768564	2.018992	Greater	0.037
	Bulk density	-0.2344647	-1.721094	Greater	0.980
	Soil water	0.6180820	6.373519	Greater	0.001

(TN, total nitrogen; TP, total phosphorus; TK, total potassium; AN, available nitrogen; AP, available phosphorus; AK, available potassium; C, carbon).

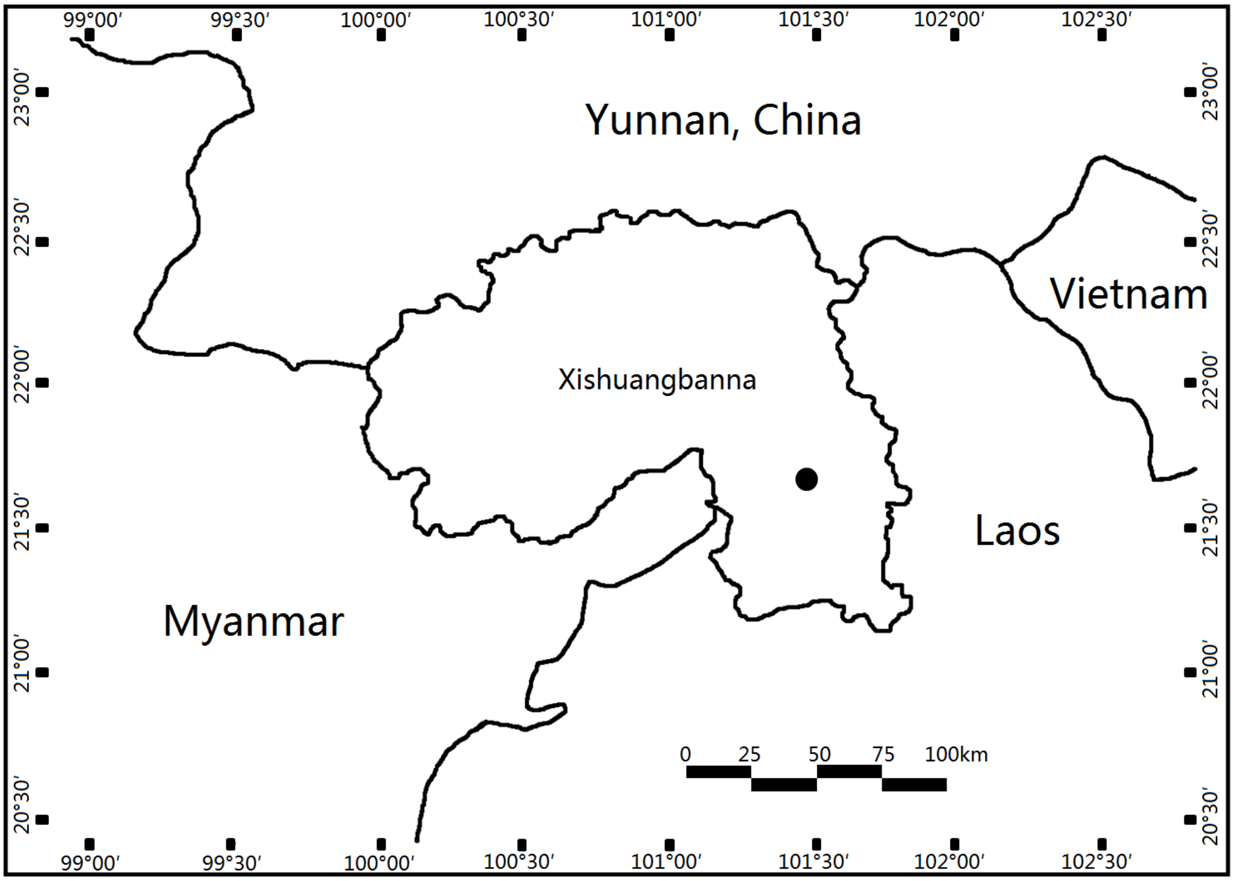

Figure S1 Geographical location map of Xishuangbanna forest dynamics plot (The black circle dot). The map was generated using ArcGIS 10.1 (www.esri.com).

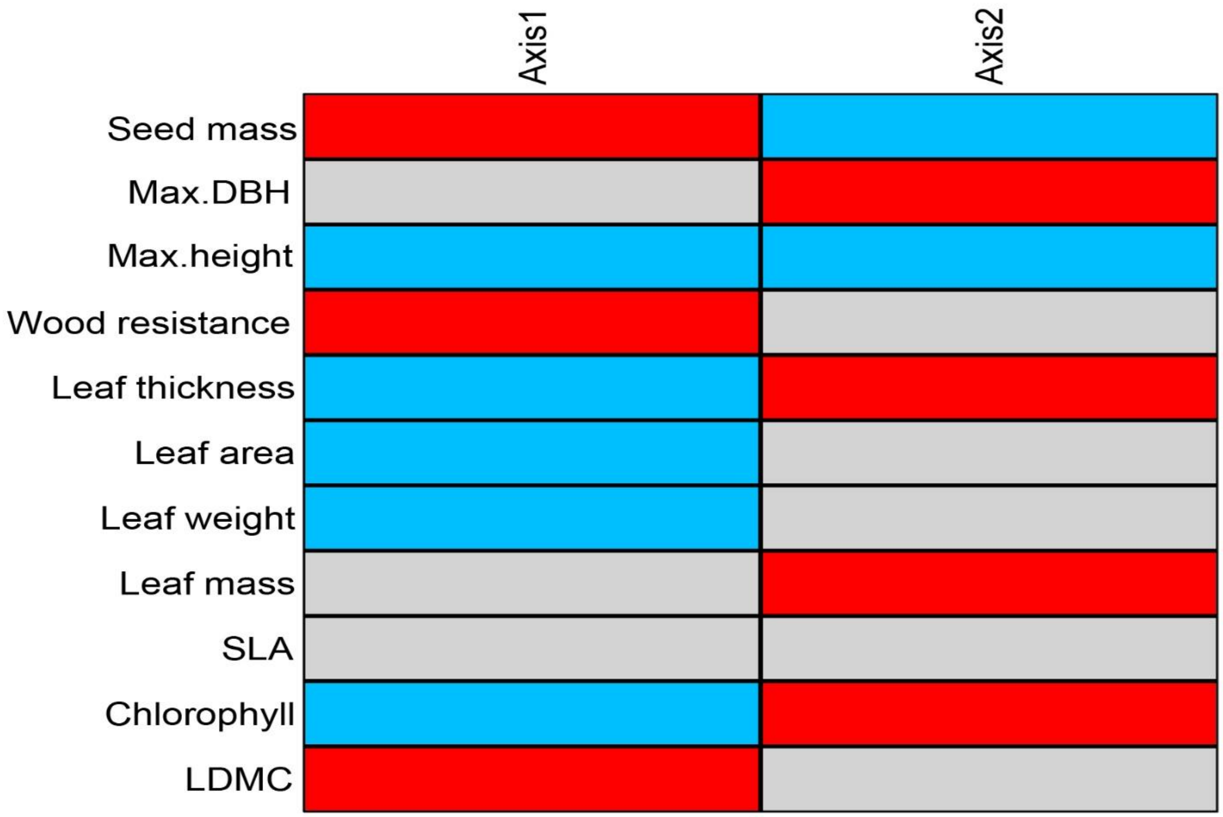

(Max. DBH, maximum diameter at breast height; Max. height, maximum height; SLA, specific leaf area; LDMC, leaf dry matter content)

Figure S2 Correlation of functional traits with the first two axes of environmental gradients using combination of fourth corner and RLQ results at the 10 × 10 m spatial scale. Significant (P, 0.05). Red cells indicate positive significant associations, blue cells indicate significant negative associations. Grey cells indicate non-significant associations.

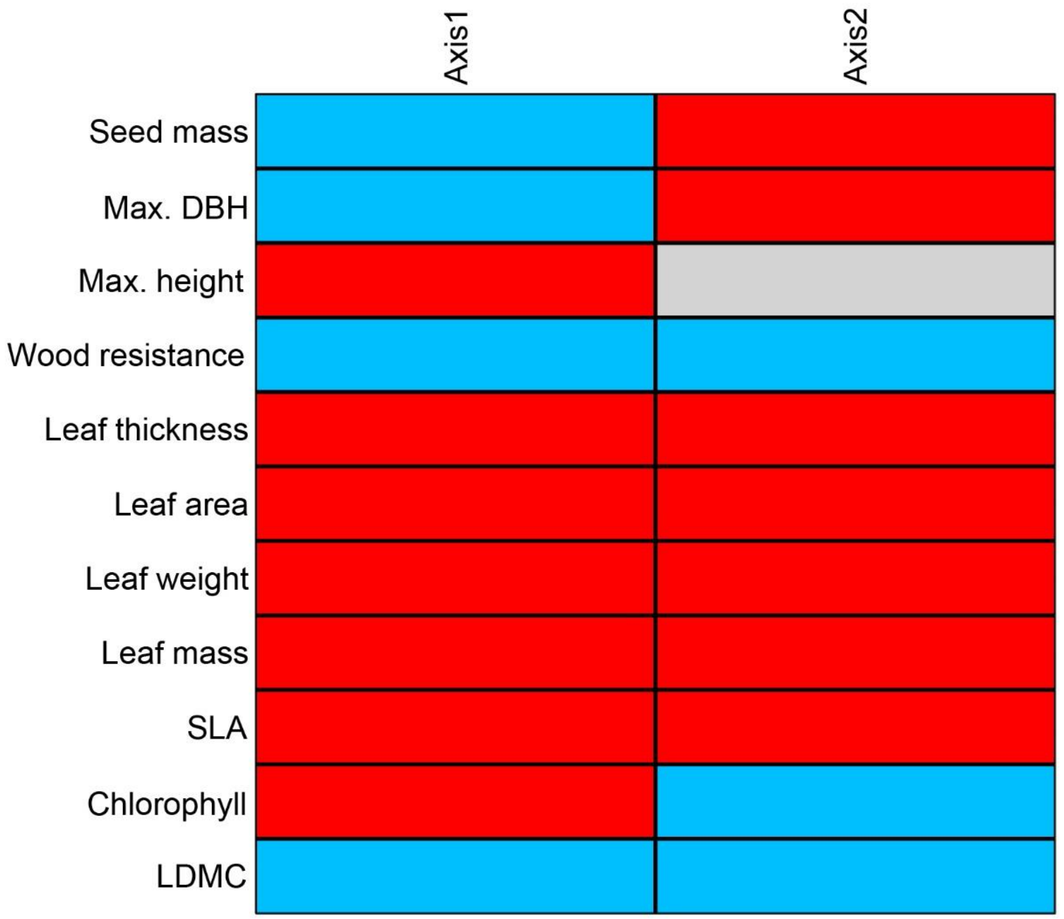

(Max. DBH, maximum diameter at breast height; Max. height, maximum height; SLA, specific leaf area; LDMC, leaf dry matter content)

Figure S3 Correlation of functional traits with the first two axes of environmental gradients using combination of fourth corner and RLQ results at the 20 × 20 m spatial scale. Significant (P, 0.05). Red cells indicate positive significant associations, blue cells indicate significant negative associations. Grey cells indicate non-significant associations.

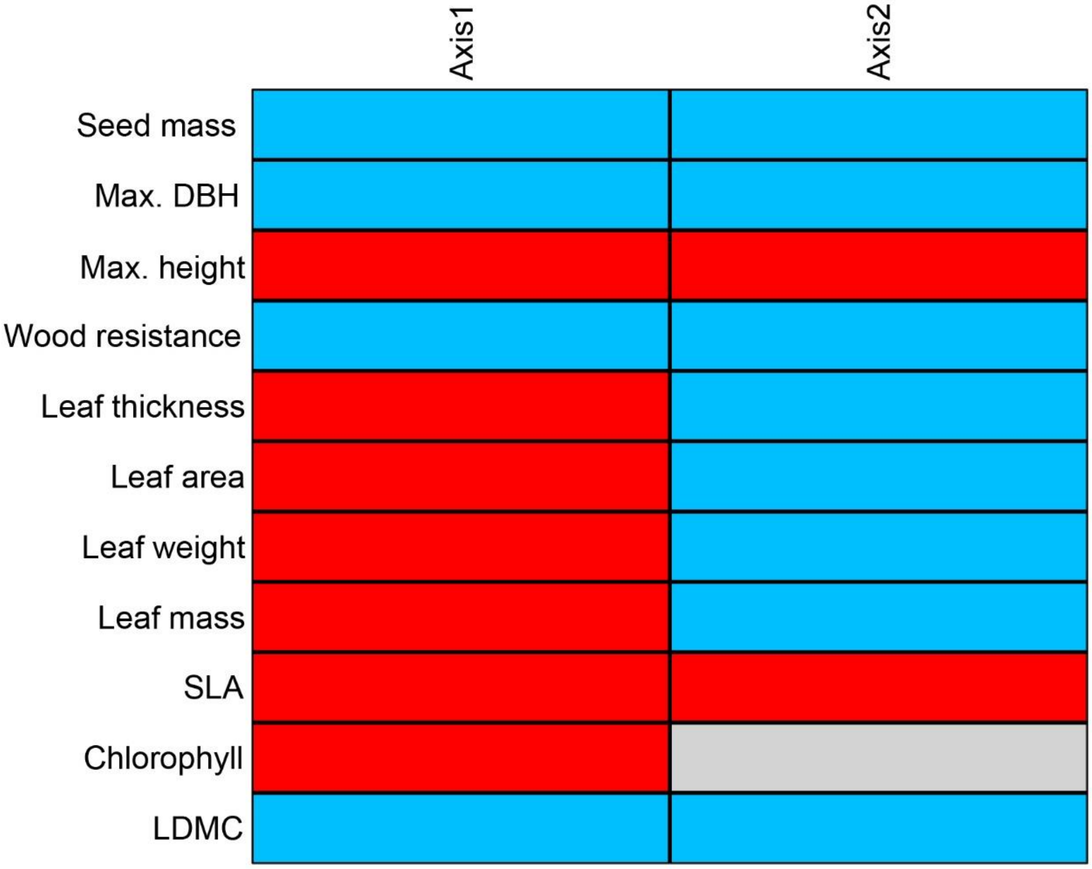

(Max. DBH, maximum diameter at breast height; Max. height, maximum height; SLA, specific leaf area; LDMC, leaf dry matter content)

Figure S4 Correlation of functional traits with the first two axes of environmental gradients using combination of fourth corner and RLQ results at the 50 × 50 m spatial scale. Significant (P, 0.05). Red cells indicate positive significant associations, blue cells indicate significant negative associations. Grey cells indicate non-significant associations.

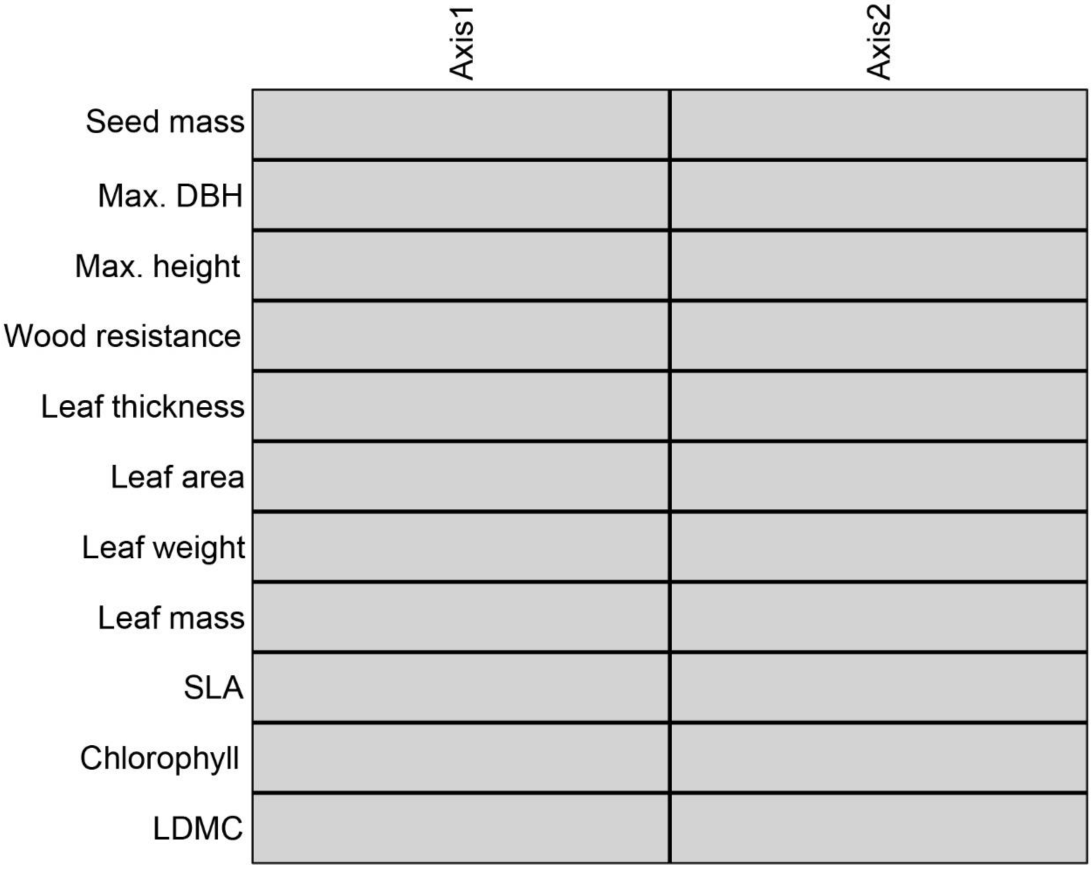

(Max. DBH, maximum diameter at breast height; Max. height, maximum height; SLA, specific leaf area; LDMC, leaf dry matter content)

Figure S5 Correlation of functional traits with the first two axes of environmental gradients using combination of fourth corner and RLQ results at the 100 × 100 m spatial scale. Grey cells indicate non-significant associations.

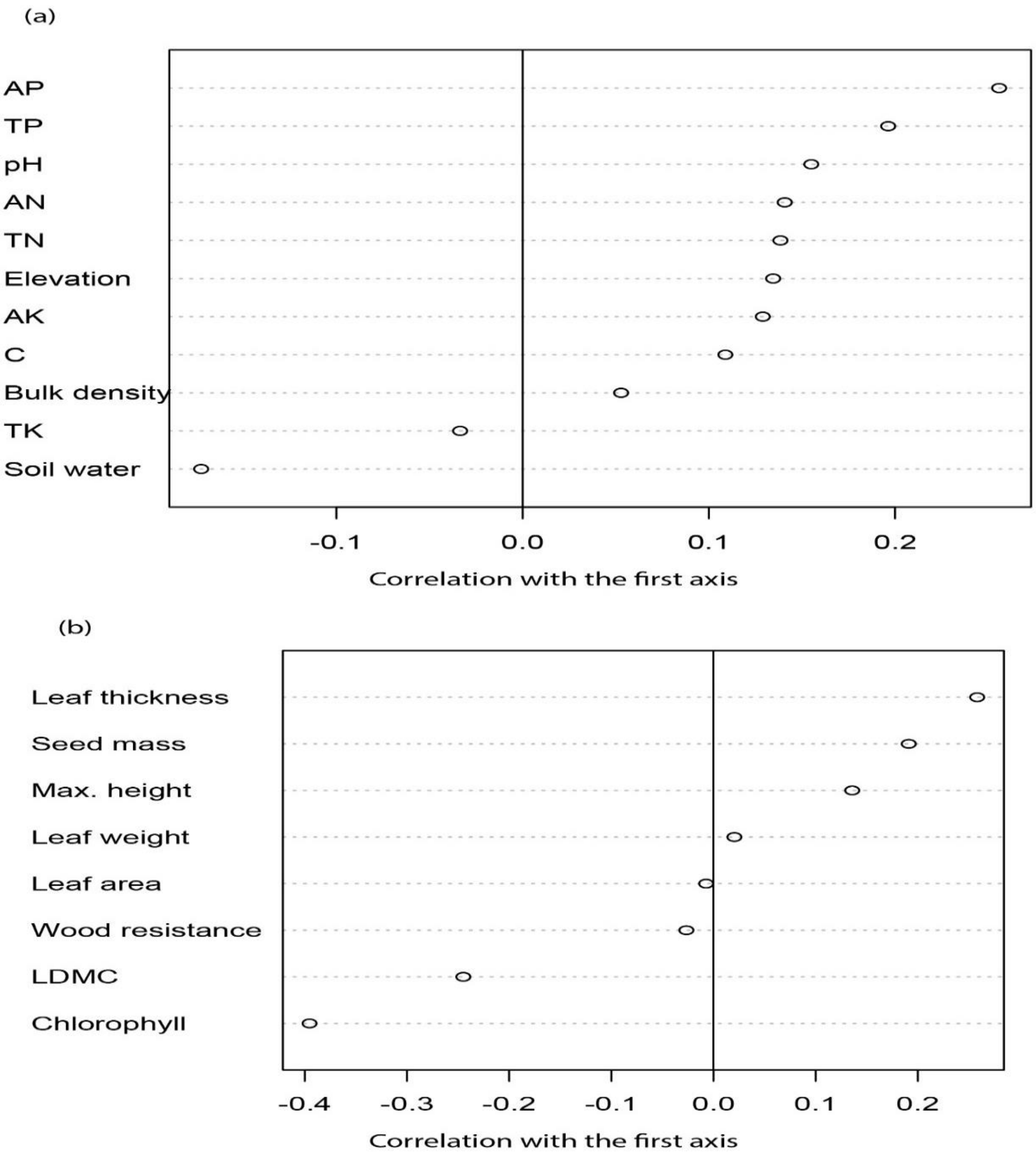

(AP, available Phosphorus; AN. available Nitrogen; TN, total Nitrogen; TP, total Phosphorus; C, carbon, AK, available Potassium; TK, total Potassium; Max. height, maximum height; LDMC, leaf dry matter content)

Figure S6 Effects of environmental variables and functional traits on the first axis of the RLQ analysis at the 10 × 10 m spatial scale. (a) Pearson correlation between environmental variables and the coordinates of sites on the first axis. (b) Pearson correlation between traits and the coordinates of species on the first axis.

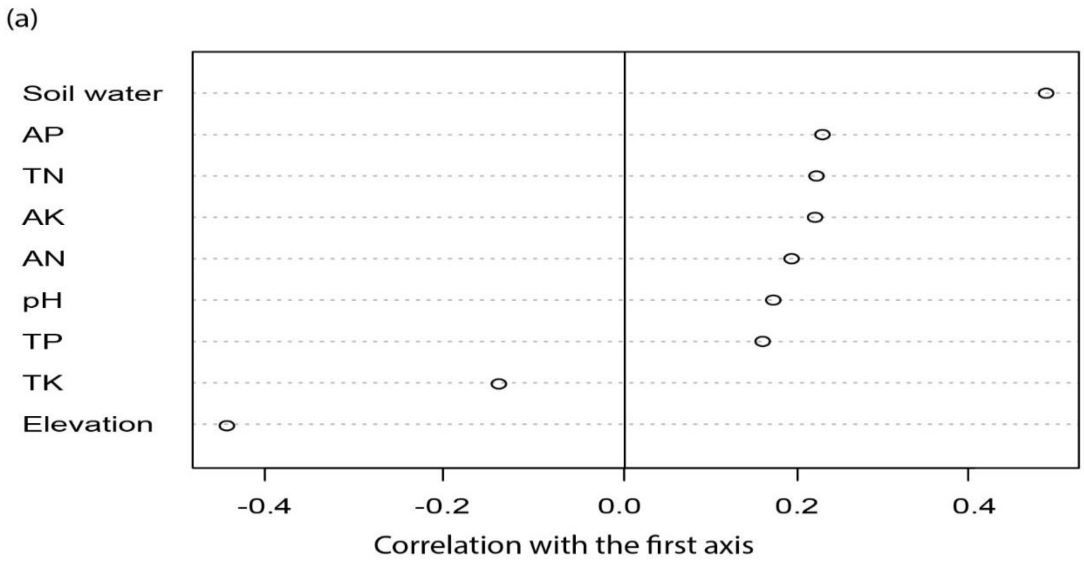

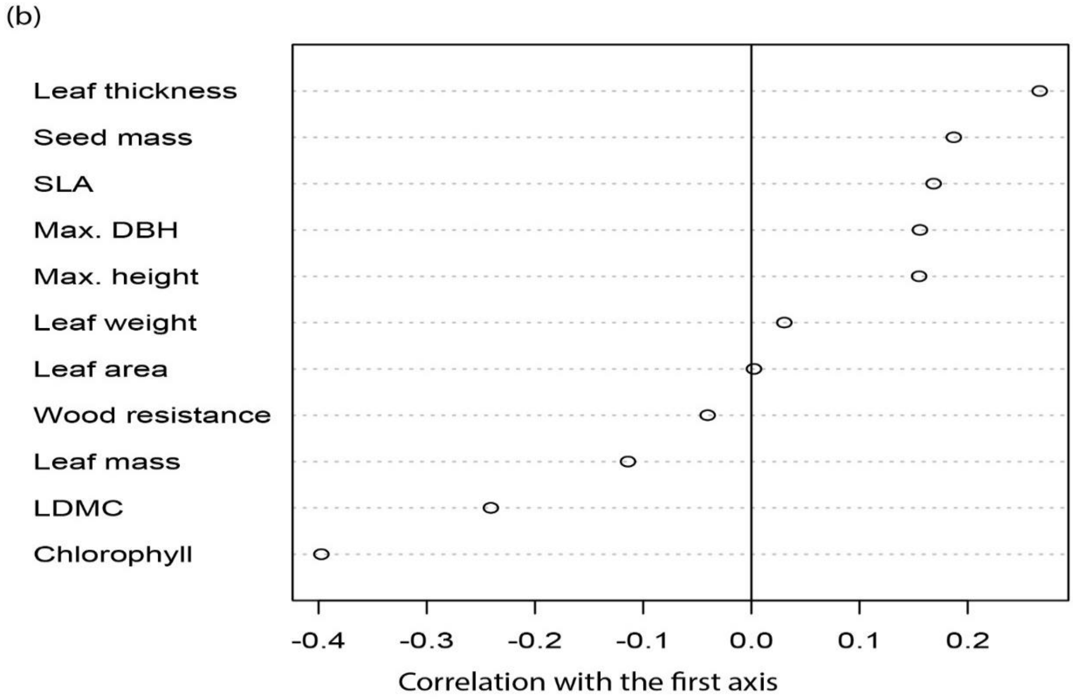

Figure S7 Effects of environmental variables and functional traits on the first axis of the RLQ analysis at the 50 × 50 m spatial scale. (a) Pearson correlation between environmental variables and the coordinates of sites on the first axis. (b) Pearson correlation between traits and the coordinates of species on the first axis.

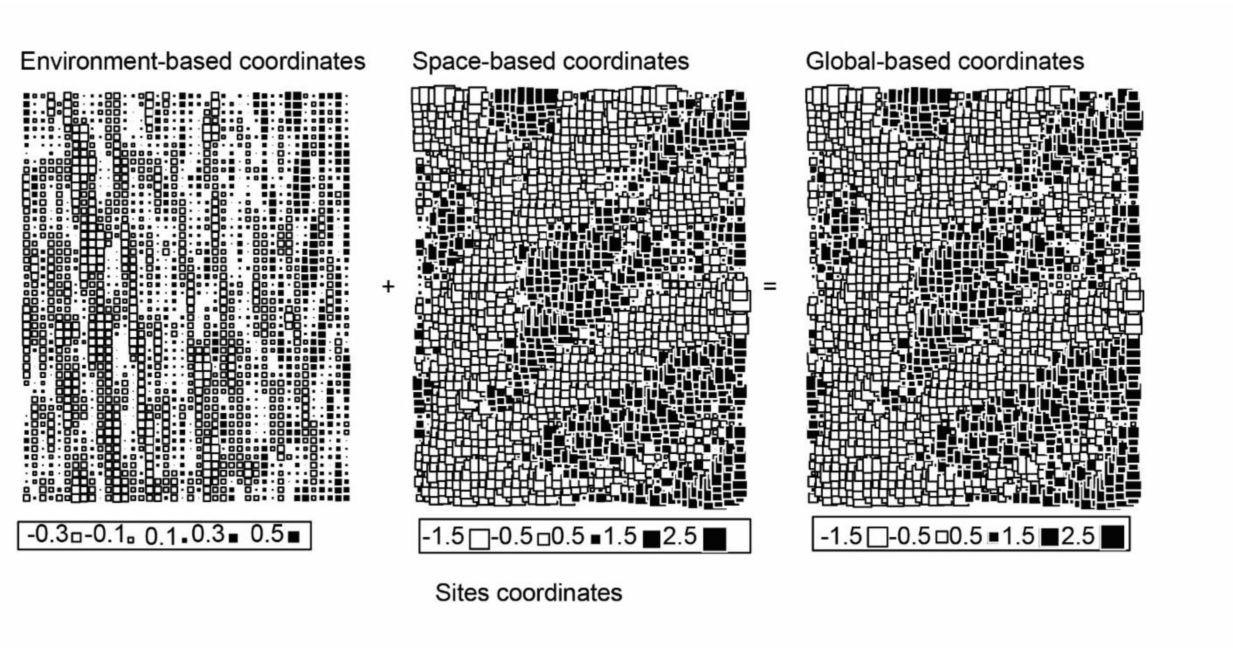

Figure S8 Result of the RLQ analysis visualized on the geographic area at 10 × 10 m. The coordinates of sites analysed on the first axis only. The global coordinates of the sites are defined as the sum of a combination of environmental variables and a combination of spatial variables. The size of the squares is proportional to the absolute values of the site coordinates; black squares indicate a positive coordinate, and white indicate a negative coordinate.

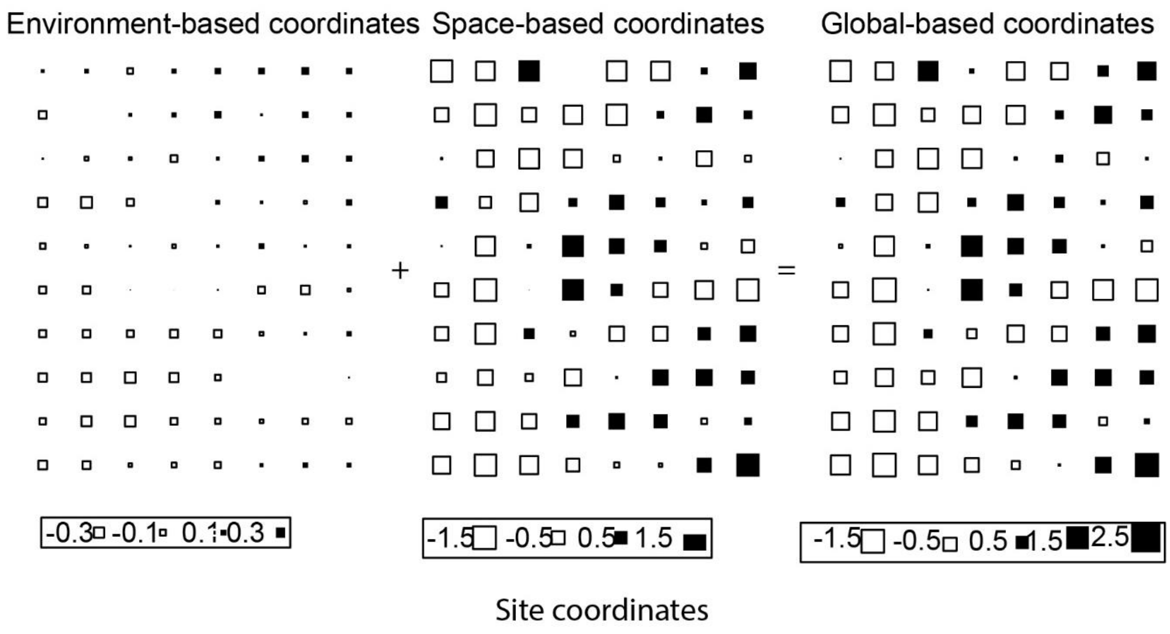

Figure S9 Result of the RLQ analysis visualized on the geographic area at 50 × 50 m. The coordinates of sites analysed on the first axis only. The global coordinates of the sites are defined as the sum of a combination of environmental variables and a combination of spatial variables. The size of the squares is proportional to the absolute values of the site coordinates; black squares indicate a positive coordinate, and white indicate a negative coordinate.

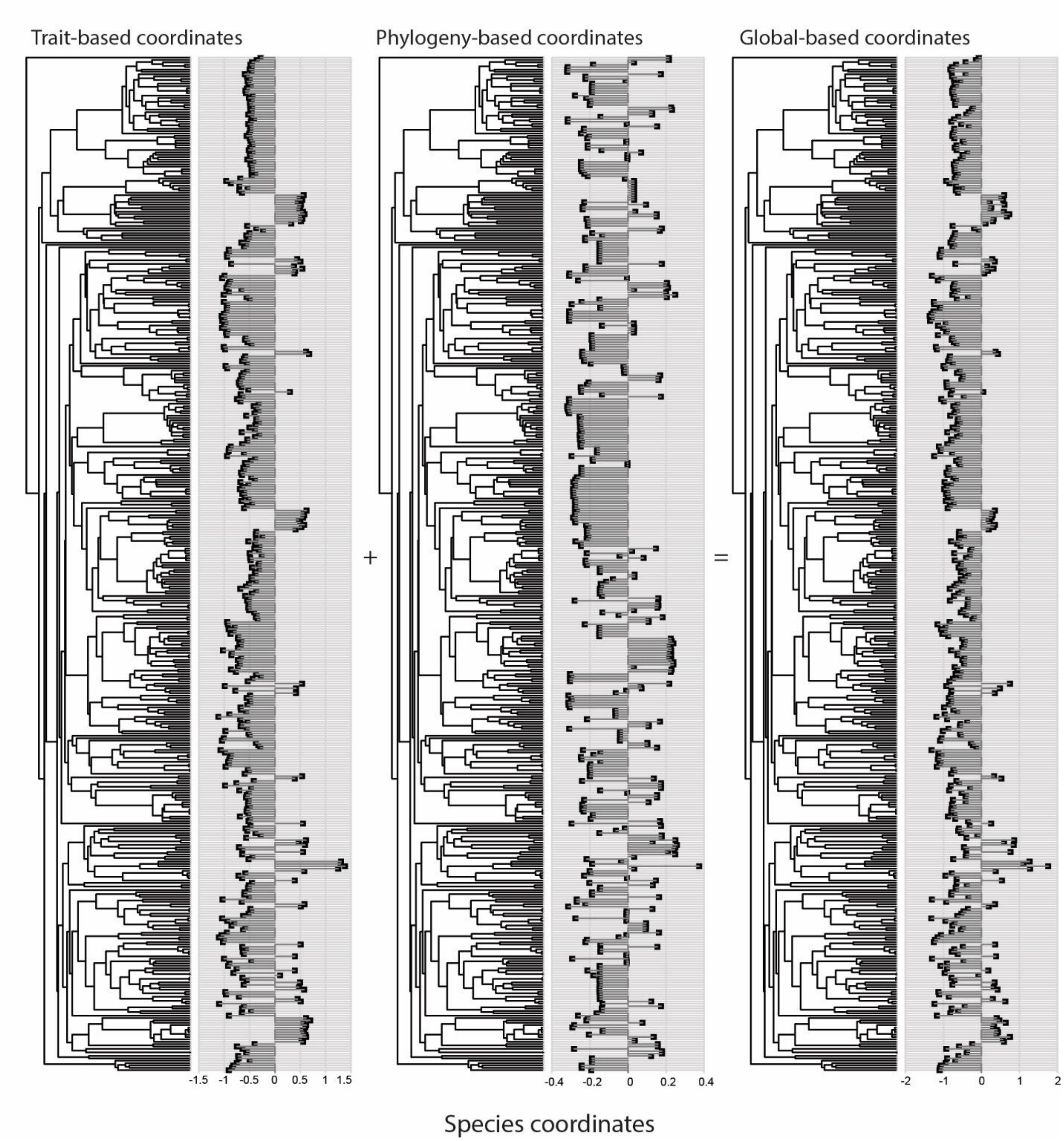

Figure S10 Result of the RLQ analysis visualized on the phylogeny at 10 × 10 m. The coordinates of species are analysed on the first axis only. The global coordinates of the species are defined as the sum of a combination of trait variables and a combination of phylogenetic variables.

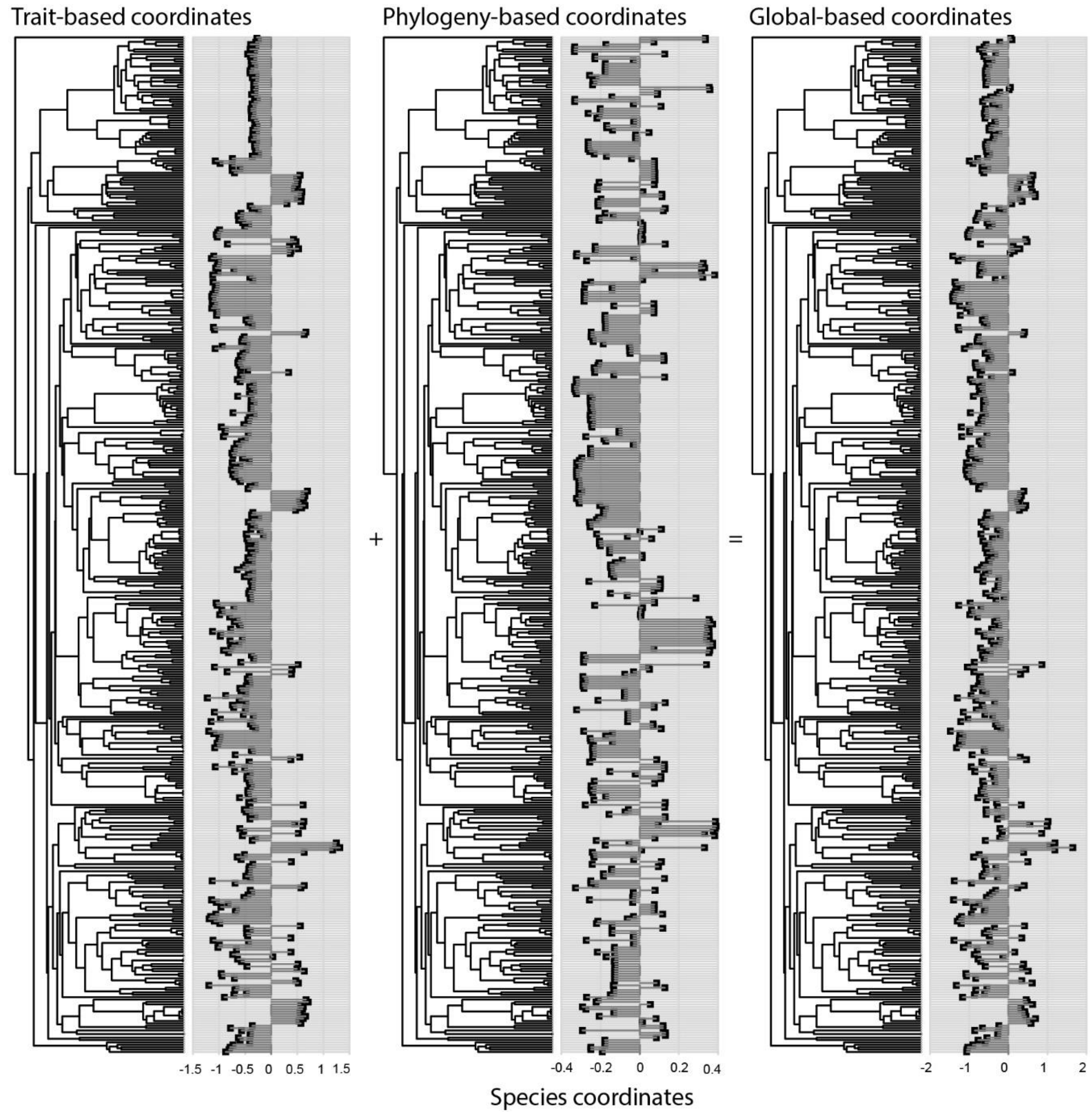

Figure S11 Result of the RLQ analysis visualized on the phylogeny at 50 × 50 m spatial scale. The coordinates of species are analysed on the first axis only. The global coordinates of the species are defined as the sum of a combination of trait variables and a combination of phylogenetic variables.
